# Author Correction: Tree rings reveal globally coherent signature of cosmogenic radiocarbon events in 774 and 993 CE

**DOI:** 10.1038/s41467-018-07636-6

**Published:** 2018-12-17

**Authors:** Ulf Büntgen, Lukas Wacker, J. Diego Galván, Stephanie Arnold, Dominique Arseneault, Michael Baillie, Jürg Beer, Mauro Bernabei, Niels Bleicher, Gretel Boswijk, Achim Bräuning, Marco Carrer, Fredrik Charpentier Ljungqvist, Paolo Cherubini, Marcus Christl, Duncan A. Christie, Peter W. Clark, Edward R. Cook, Rosanne D’Arrigo, Nicole Davi, Ólafur Eggertsson, Jan Esper, Anthony M. Fowler, Ze’ev Gedalof, Fabio Gennaretti, Jussi Grießinger, Henri Grissino-Mayer, Håkan Grudd, Björn E. Gunnarson, Rashit Hantemirov, Franz Herzig, Amy Hessl, Karl-Uwe Heussner, A. J. Timothy Jull, Vladimir Kukarskih, Alexander Kirdyanov, Tomáš Kolář, Paul J. Krusic, Tomáš Kyncl, Antonio Lara, Carlos LeQuesne, Hans W. Linderholm, Neil J. Loader, Brian Luckman, Fusa Miyake, Vladimir S. Myglan, Kurt Nicolussi, Clive Oppenheimer, Jonathan Palmer, Irina Panyushkina, Neil Pederson, Michal Rybníček, Fritz H. Schweingruber, Andrea Seim, Michael Sigl, Olga Churakova (Sidorova), James H. Speer, Hans-Arno Synal, Willy Tegel, Kerstin Treydte, Ricardo Villalba, Greg Wiles, Rob Wilson, Lawrence J. Winship, Jan Wunder, Bao Yang, Giles H. F. Young

**Affiliations:** 1 Department of Geography, University of Cambridge, Cambridge CB2 3EN, UK; 2 Swiss Federal Research Institute WSL, CH-8903 Birmensdorf, Switzerland; 3 Global Change Research Institute CAS, 603 00 Brno, Czech Republic; 4 Department of Geography, Masaryk University, 611 37 Brno, Czech Republic; 5 Laboratory for Ion Beam Physics, ETH Zürich, CH-8093 Zurich, Switzerland; 6 Département de Biologie, Chimie et Géographie, University of Québec in Rimouski, QC G5L 3A1 Canada; 7 School of Natural and Built Environment, Queen’s University, Belfast BT7 1NN, Northern Ireland UK; 8 Swiss Federal Institute of Aquatic Science and Technology Eawag, CH-8600 Dübendorf, Switzerland; 9 CNR-IVALSA, Trees and Timber Institute, 38010 San Michele all′Adige, TN Italy; 10 Competence Center for Underwater Archaeology and Dendrochronology, Office for Urbanism, City of Zurich, 8008 Zürich, Switzerland; 11 School of Environment, University of Auckland, 1010 Auckland, New Zealand; 12 Institute of Geography, Friedrich-Alexander-University Erlangen-Nürnberg (FAU), 91058 Erlangen, Germany; 13 Department Territorio e Sistemi Agro-Forestali, University of Padova, 35020 Legnaro (PD), Italy; 14 Department of History, Stockholm University, SE-10691 Stockholm, Sweden; 15 Bolin Centre for Climate Research, Stockholm University, SE-10691 Stockholm, Sweden; 16 Laboratorio de Dendrocronología y Cambio Global, Universidad Austral de Chile, Casilla 567, Valdivia, Chile; 17 Center for Climate and Resilience Research, Blanco Encalada 2002, 8370449 Santiago, Chile; 18 Rubenstein School of Environment and Natural Resources, University of Vermont, Burlington, Vermont 05405, USA; 19 Tree-Ring Laboratory, Lamont-Doherty Earth Observatory of Columbia University, Palisades, NY 10964-8000 USA; 20 Department of Environmental Science, William Paterson University, Wayne, NJ 07470 USA; 21 Icelandic Forest Research Mógilsá, 116 Reykjavik, Iceland; 22 Department of Geography, Johannes Gutenberg University, 55099 Mainz, Germany; 23 Department of Geography, University of Guelph, ON N1G 2W1 Canada; 24 AgroParisTech, INRA, Université de Lorraine, 54000 Nancy, France; 25 Department of Geography, University of Tennessee, Knoxville, TN 37996-0925 USA; 26 Swedish Polar Research Secretariat, SE-104 05, Stockholm, Sweden; 27 Department of Physical Geography, Stockholm University, SE-106 91 Stockholm, Sweden; 28 Institute of Plant and Animal Ecology, Ural Branch of the Russian Academy of Sciences, Ekaterinburg 620144, Russia; 29 Bavarian State Office for Monument Protection, 80539 München, Germany; 30 Department of Geology and Geography, West Virginia University, WV 26505-6300 USA; 31 German Archaeological Institute, 14195 Berlin, Germany; 32 Department of Geosciences, University of Arizona, Tucson, AZ 85721 USA; 33 AMS Laboratory, University of Arizona, Tucson, AZ 85721 USA; 34 Isotope Climatology and Environmental Research Centre, Institute of Nuclear Research, H-4001 Debrecen, Hungary; 35 Sukachev Institute of Forest SB RAS, 660036 Krasnoyarsk, Russia; 36 Department of Humanities, Siberian Federal University, 660041 Krasnoyarsk, Russia; 37 Department of Wood Science, Mendel University in Brno, 61300 Brno, Czech Republic; 38 Navarino Environmental Observatory, GR-24001 Messinia, Greece; 39 Department of Earth Sciences, University of Gothenburg, 405 30 Gothenburg, Sweden; 40 Department of Geography, Swansea University, Swansea SA2 8PP, Wales UK; 41 Department of Geography, University of Western Ontario, London, ON N6A 3K7 Canada; 42 Institute for Space-Earth Environmental Research, Nagoya University, Nagoya 464-8601, Japan; 43 Institute of Geography, University of Innsbruck, 6020 Innsbruck, Austria; 44 Palaeontology, Geobiology and Earth Archives Research Centre, and ARC Centre of Excellence for Australian Biodiversity and Heritage, School of Biological, Earth and Environmental Sciences, The University of New South Wales, Sydney, NSW 2052 Australia; 45 Laboratory of Tree-Ring Research, University of Arizona, Tucson, AZ 85721 USA; 46 Harvard Forest, Harvard University, Petersham, MA 01366 USA; 47 Chair of Forest Growth and Dendroecology, Institute of Forest Sciences, University of Freiburg, Freiburg, Germany; 48 Laboratory of Environmental Chemistry, Paul Scherrer Institute, 5232 Villigen, Switzerland; 49 Institute for Environmental Sciences, University of Geneva, 1205 Geneva, Switzerland; 50 Department of Earth and Environmental Systems, Indiana State University, Terre Haute, IN 47809 USA; 51 Archaeological Service Kanton Thurgau (AATG), 8510 Frauenfeld, Switzerland; 52 Instituto Argentino de Nivología, Glaciología y Ciencias Ambientales, IANIGLA–CONICET, Mendoza, CP 330 5500 Argentina; 53 Department of of Earth Sciences, The College of Wooster, OH 44691 USA; 54 School of Geography and Geosciences, University of St Andrews, St Andrews KY16 9AJ, Scotland UK; 55 School of Natural Science, Hampshire College, Amherst, MA 01002 USA; 56 Key Laboratory of Desert and Desertification, Northwest Institute of Eco-Environment and Resources, Chinese Academy of Sciences, 730000 Lanzhou, China

Correction to: *Nat. Commun.* 10.1038/s41467-018-06036-0; published online 6 Sep 2018.

The original version of this Article contained an error in the Data Availability section, which incorrectly read ‘All data will be freely available via https://www.ams.ethz.ch/research.html.’ The correct version states ‘http://www.ams.ethz.ch/research/published-data.html’ in place of ‘https://www.ams.ethz.ch/research.html’. This has been corrected in both the PDF and HTML versions of the Article.

