# Supplementary material for: Tree rings reveal globally coherent signature of cosmogenic radiocarbon events in 774 and 993 CE
Source: Nat Commun. 2018 Sep 6;9:3605. doi: 10.1038/s41467-018-06036-0 (PMC6127282; doi:10.1038/s41467-018-06036-0)
Supplement: Supplementary file 1 — Supplementary Information [file 41467_2018_6036_MOESM1_ESM.pdf]

Supplementary Information for **Tree rings reveal globally coherent signature of cosmogenic radiocarbon events in 774 and 993 CE** *by* U. Büntgen *et al.*

Supplementary Note 1: Tree-ring network

The dendrochronologically cross-dated tree-ring samples in our COSMIC network were collected over several decades, for a variety of purposes, and represent enormously different environmental and climatic settings around the globe (Fig. 1; Supplementary Fig. 1; Supplementary Tables 1-2). Most were collected for the explicit purpose of dendroclimatological reconstruction of past temperature or hydroclimate. The network includes material from high, medium, and low elevation sites between around 40 and 4000 m asl on five continents. The network's climatic conditions range from sub-Arctic forest-tundra at ~72° N to patches of woodland growing on the edge of deserts in Northern and Southern America, the European Mediterranean and different parts of inner Eurasia and Asia. The only environmental/climatic settings missing in our dataset are the tropics, owing to the difficulties to conduct annual precise cross-dated dendroclimatological research there (see also details below), as well as the real polar and alpine climate zones where trees do not grow. In consideration of this global sample distribution, the coherent cosmic event signatures detailed herein are even more remarkable. A brief description of the COSMIC tree-ring network, subdivided into eight clusters (North America, South America, Scandinavia, central and southern Europe, northern Siberia, inner Eurasia, Monsoon Asia and Australasia), and complementing the information in Supplementary Tables 1–2, follows.

North America (United States and Canada): USA10 comes from a Mountain hemlock (*Tsuga mertensiana*) sample from the Great Nunatak mountain region at the head of the Columbia Bay fjord in the Prince William Sound[1], Alaska. This site represents the very northernmost point of the

coastal Pacific temperate rain forest where climate conditions are more sub-Arctic boreal than the mild oceanic temperate climate typical for the biome. USA11, also Mountain hemlock from the coastal Pacific temperate rain forest in the Gulf of Alaska[1], represents a low-elevation, distinctly non-boreal ecotype, more typical of mid-latitudes but with strong oceanic influences, producing cool summers and (for the latitude) relatively mild winters.

CAN06 comes from black spruce (*Picea mariana*) collected at the transition area between open lichen woodland and the forest-tundra of the Québec-Labrador Peninsula in eastern Canada[2]. This region exhibits the most Arctic-like climate conditions in the world at such a southern latitude owing to the strong cooling influences of the Labrador Sea to the east and the Hudson Bay to the west. At ~54° N the climate is sub-Arctic and the site is located within the discontinuous permafrost zone. The monthly mean daily temperature in July and August reaches up to only ~14° C. The tree-ring material was collected from subfossil trees preserved in lakes for the explicit purpose of creating a summer temperature reconstruction.

USA16 comes from a Ponderosa pine (*Pinus ponderosa* Dougl. ex Laws.) collected at the Moskt Butte lava flow in Newberry Crater National Monument in central Oregon[3], United States. Trees at this site were growing at an elevation of ~1500 m asl. At this elevation the growing season is less than 60 days. Despite the rather cold conditions at this elevation, the site is considered semi-arid in the rain shadow of the Cascade Mountains. The climate signal of the trees is mainly hydroclimatic. USA18 comes from a very high-elevation, bristlecone pine (*Pinus longaeva*) site in the Sheep Mountains[4], California, United States, in the Sierra Nevada range near the Nevada state boundary. The environment is both dry and cool, and the climate signal from the site is hard to constrain. Bristlecone pine chronologies from this region have been used to infer both, past hydroclimate as well as temperature variability.

USA07 and USA02 represent Douglas-fir (*Pseudotsuga menziesii*) chronologies from the El Malpais National Monument[5], New Mexico, United States. The region is a volcanically active area on the southeast margin of the Colorado Plateau by the intersection of the Rio Grande Rift Basin. The climate is semi-arid with steppe and half-desert environments at lower elevations, and open grasslands and forests at overall higher elevations. The tree-ring chronology, consisting of living and dead material, was originally developed to reconstruct wildfire histories and their short- and long-term relationships with climate.

South America (Chile): PAT02 comes from El Asiento in central Chile and represents material from precipitation sensitive Chilean cedar (*Austrocedrus chilensis*) growing on steep rocky slopes of the Andes near the species' northern distribution limit[6]. The trees were collected with the purpose to make a quantitative precipitation reconstruction. The climate in the region is on the border between cool semi-arid and Mediterranean; winters (June–August) are fairly cool and humid and summers (November–March) are rather warm and dry. Annual precipitation totals are between 300 and 400 millimetres, resulting in moisture stress during the dry summer season for the trees. PAT03 comes from Patagonian cypress (*Fitzroya cupressoides*) from Quildaco in the Los Lagos Region of Chile[7]. This site is typical for the Valdivian temperate rain forest region with a mild (maritime) climate receiving an annual precipitation of 4,000-4,500 mm concentrated in SH winter (June-August).

Scandinavia (Norway and Sweden): FOR01 comes from Scots pine (*Pinus sylvestris* L.) in Forfjorddalen[8], a valley located on Andoya, one of the innermost islands of the mountainous archipelago Lofoten in northern Norway just above the Arctic Circle. The climate is subpolar-oceanic, with surprisingly mild winters and cold summer, relative to the more continental conditions of much of the northern boreal zone. The samples come from fallen or standing dead

trees. SWE01, SWE02, and SWE04 also represent relict Scots pines from the two sites in Alajärvi[9] and Gaisenjarka[10] in the Torneträsk area of the northern boreal zone in northernmost Sweden. The region is situated in the Scandes mountains close to the regional northern and upper treeline, where the overall sub-Arctic climate is slightly moderated by the maritime influences of the North Atlantic. SWE05 and SWE02 are further Scots pine samples from Håckervalen[11], Jämtland in north-central Sweden, located in the northern boreal zone. All of the Swedish and Norwegian sites are in general outside the permafrost region although some discontinues permafrost exists in the area of Lake Torneträsk in northern Sweden.

Central and southern Europe (Germany, Austria, Switzerland, Italy, Albania and Greece): GER01 comes from oak (*Quercus spp*) from the river Main in the area of Aschaffenburg[30 (main text)], Germany. Under present-day climate conditions, oak growth in this region is mainly limited by spring to early-summer precipitation totals. Likewise, the GER07 (unpublished) from an archaeological excavation in Plattling, Bavaria, Germany, coming from Silver fir (*Abies alba*), represents a more drought-prone species and environment. KOFL06 comes from Swiss stone pine (*Pinus cembra*) at a south-facing slope close to the upper treeline at 2165–2190 m asl[12,13], on Kofler Alm in South Tyrol, Italy. This region belongs to the western section of the central Eastern Alps, and fieldwork between 1998 and 2006 mainly focussed on subfossil logs in peat bogs and small lakes. Growing season temperature is the main limiting factor for tree growth at this high-elevation, central European site. EBA101, also of Swiss stone pine, comes from Eben Alm[9 (main text),12,13], another, similar high-elevation site in Tyrol. SUI01 comes from European larch (*Larix decidua*) that was sampled in a well-preserved, wooden roof construction of the Holy Cross Chapel[11 (main text)], Convent St. John the Baptist in Val Müstair, Switzerland. Collected for the purpose of dendrochronological dating, the exact climatological response of the trees, although

assumed to be warm season temperature limited, remains unknown. ITA09 (unpublished) comes from Norway spruce (*Picea abies*) subfossil wood, preserved in high-elevation peat bogs in the Trentino region of the Italian Alps. The climate of this region is considered relatively cold sub-continental, with winter and summer temperatures ranging between around −12° C to −5° C and 12° C, respectively. Tree growth is expected to be mainly temperature limited.

ALB01 represents material from a dead Bosnian pine (*Pinus heldreichii*) found within an open upper forest ecotone of Fushe Lura in central Albania[14]. The tree growth–climate relationship is complex, and like other tree-ring records from this region, characterised by a mixed hydroclimate and temperature signal. GRE02, also Bosnian pine, comes from Mount Smolikas in the Pindos National Park in northern Greece[15]. This area, considered an upper oro-mediterranean vegetation zone, is barren and steppe like, with scattered patches of pure stands of and little or no undergrowth. The exposed bedrock is serpentine and poor in nutrients. At over 2000 m asl, the winters are hard and snow cover persists well into spring. Dry summer conditions are mainly restricted to June–August, and frequently interrupted by summer storms with convective rainfall.

Northern Siberia (Russia): RUS04 originates from subfossil Siberian larch (*Larix sibirica*) wood in alluvial deposits of the Yadayakhodyyakha River in the Yamal Peninsula of northwestern Siberia[10 (main text),16-18]. RUS20 also comes from Siberian larch from a slightly more northern part of the Yamal Peninsula[10 (main text),16-18], though representing similar conditions. Both sites are located in the continuous permafrost zone in the taiga-tundra ecotone. Summers are not as warm as further east at similar latitudes in the interior of Siberia and, consequently, the treeline does not extend much further north of the Arctic Circle. RUS15 is of Dahurian larch (*Larix gmelinii* Rupr.) from the Kotuy River on the eastern Taimyr Peninsula in northern Siberia[19]. This region, located in the continuous permafrost zone, is home to the most northern trees in the world (>72° N) due

to an extreme continental climate (with a July monthly mean temperature >12° C). Lakes and rivers are frozen from early October until well into June. The average annual growing season is possibly the shortest in the world for a forested area (according to nearby instrumental observations, 50-70 days have mean temperatures >5° C). RUS17 (unpublished) comes from Cajander larch (*Larix cajanderi* Mayr.) from the Indigirka River in Yakutia, northeastern Siberia[20,21], east of the Kusagan-Mastach Mountains at 200-350 m asl. Like the other northern Siberian tree-ring sites, the region is situated in the continuous permafrost zone of the taiga-tundra ecotone. Moreover, northeastern Yakutia has an extremely continental climate with very cold winters and chilly summers, often ranging from monthly means well below –38° C in January to warmer than 9° C in July (measured at the nearby weather station in Chokurdach). Moreover, annual precipitation totals are with ~205 mm/year very low.

Inner Eurasia (Russia, Mongolia and Pakistan): ALT01 and ALT02 comes from Siberian larch (*Larix sibirica* Ledeb) from high-elevation sites[3 (main text),22], close to the upper treeline, in the Altai Mountains of Russia close to the borders of China, Mongolia, and Kazakhstan. The ecotone is a mixture of steppes, boreal forest (taiga), and alpine vegetation. The samples represent the regional upper treeline ecotone ~2300 m asl in the Tuva Province where *in situ* relict larch trees are abundant. MON09 comes from Siberian pine (*Pinus sibirica*) from the Khorgo lava field in central Mongolia[23]. This material was collected in 2010 and 2012 from living and dead trees on thin or absent soils surrounded by dark basalt. The site is above 2000 m asl where a pronounced continental climate is further characterised by cold summers. MON03 and MON05 (unpublished) come from an equally harsh environment at the Uurgat site in the Övörkhangai region[24], south and east of Khorgo. Water stress is the main limiting factor at all Mongolian sites, but there is also a likely temperature component. PAK04 comes from the Satpara Valley[25], Pakistan. Very slow

growing Greek juniper (*Juniperus excelsa*) trees were samples at a high-elevation, dry environment ~3700 m asl. Both growing season temperature means and soil moisture availability are growth limiting factors.

Monsoon Asia (China and Japan): CHI01 comes from Qilian juniper (*Juniperus przewalskii* Kom.) in the Delingha region in the eastern Qaidam Basin[26], China. The site is located at the present-day edge of the Asian summer monsoon region. The elevation exceeds 4000 m asl and the climate is dry and cold. Forested areas are restricted to certain locations and the landscape bears significant imprints of anthropogenic activities. Despite relatively cold conditions, the main limiting factor in most of the region is drought. TIB01 represents Tibetan juniper (*Juniperus tibetica*) from the Zhujiao Mountains in the northwestern part of Qamdo County[27], on the south-eastern Tibetan Plateau, China. The environmental setting is open woodland in a relatively cold environment >4000 m asl. The location is not as dry as many other parts of the Tibetan Plateau, with up to 500 millimetres precipitation per year, and a monthly daily mean temperature range from about –5° C in January to +14° C in July. Nearly 80% of the annual precipitation falls during the growing between May and September. JAP01 comes Japanese red cedar (*Cryptomeria japonica*) from the mountainous Yakushima Island in the southern Japanese archipelago[28], Japan. The subtropical climate is warm and humid. Although water is not expected to be a major limiting growth factor for this species and site, a mixed temperature and hydroclimatic signal is possible.

Australasia (Tasmania and New Zealand): TAS01 (unpublished) is a Huon pine (*Lagarostrobos franklinii*) sample collected during excavations conducted in the 1980s and 1990s at low elevation along the Stanley River, a tributary of the Pieman River on the west coast region of Tasmania. The region has a mild oceanic climate with ample of precipitation (>2000 mm/yr), with a natural vegetation of mainly temperate rainforest. DAR01, DAR02, DAR06 and DAR07

represent kauri wood (*Agathis australis*) from Northland[29], upper North Island of New Zealand. The material is taken from well preserved swamp logs. The region is characterised by a temperate, oceanic climate bordering on humid sub-tropical conditions. Strong marine influences result in a small temperature range throughout the year: no month during the year has an average daily mean temperature below 10° C or above 20° C. Average annual precipitation totals exceed 1000 millimetres. A mixed kauri-tairare forest was present in the Dargaville region before human arrival. Gum-land scrub vegetation, described by Europeans in the 1830s, may be a legacy of Maori burning. NEW01 (unpublished) comes from a New Zealand Silver pine (*Manoao colensoi*) buried on Weka Farm in a peat bog. Located at the west coast of New Zealand's Southland >42° S, this is the most southern site of the COSMIC network.

Supplementary Table 1. Tree-ring network. Description of the COSMIC tree-ring data sorted from north-west to south-east (No. = 1–44).

No	Code	Country	Region	Site	Longitude	Latitude	m asl.	Key publication
1	USA10	USA	Alaska	Great Nunatak	147°03' W	61°07' N	203	(*1*)
2	USA11	USA	Alaska	Glacier Bay	136°28' W	58°38' N	425	(*1*)
3	CAN06	Canada	Quebec	Caniapiscau	70°24' W	54°28' N	537	(*2*)
4	USA16	USA	Oregon	Moskt Butte	121°21' W	43°53' N	1500	(*3*)
5	USA18	USA	California	Sheep Mountains	118°44′W	37°77′N	3539	(*4*)
6	USA07	USA	New Mexico	El Malpais	108°06' W	35°57' N	2357	(*5*)
7	USA02	USA	New Mexico	El Malpais	108°06' W	35°57' N	2357	(*5*)
8	PAT02	Chile	Central Chile	El Asiento	70°49' W	32°39' S	1980	(*6*)
9	PAT03	Chile	Los Lagos	Quildaco	72°41' W	41°54' S	783	(*7*)
10	PAT03	Chile	Los Lagos	Quildaco	72°41' W	41°54' S	783	(*7*)
11	FOR01	Norway	Lofoten	Forfjorddalen	15°44' E	68°48' N	120	(*8*)
12	SWE01	Sweden	Torneträsk	Gaisenjarka	19°27' E	68°16' N	425	(*10*)
13	SWE02	Sweden	Torneträsk	Alajarvi	19°37' E	68°15' N	400	(*9*)
14	SWE04	Sweden	Torneträsk	Alajarvi	19°37' E	68°15' N	400	(*9*)
15	SWE05	Sweden	Jämtland	Håckervalen	13°34' E	63°09' N	530	(*11*)
16	SWE02	Sweden	Jämtland	Håckervalen	13°34' E	63°09' N	530	(*11*)
17	GER01	Germany	Aschaffenburg	Steinbach	08°09' E	48°43' N	149	(*30 main text*)
18	GER07	Germany	Bavaria	Plattling Pankofen	12°54' E	48°48' N	320	unpublished
19	KOFL06	Italy	South Tyrol	Kofler Alm	12°06' E	46°57' N	2170	(*13*)
20	EBA101	Austria	Tyrol	Eben Alm	10°57' E	47°01' N	2100	(*13*)
21	SUI01	Switzerland	Müstair	Kloster St. Johann	10°26' E	46°37' N	1250	(*11 main text*)
22	ITA09	Italy	Trentino, Brez	Palù Avert	11°02' E	46°18' N	1805	unpublished
23	ALB01	Albania	Central Albania	Fushe Lura	20°14' E	41°48' N	1900	(*14*)
24	GRE02	Greece	Pindos NP	Mount Smolikas	20°58' E	40°06' N	2146	(*15*)
25	RUS04	Russia	Yamal	Yadayakhodyyakha	70°42' E	67°29' N	36	(*16*)
26	RUS20	Russia	Yamal	Yadayakhodyyakha	70°40' E	67°31' N	38	(*16*)
27	RUS15	Russia	Taimyr	Kotuy River	102°38' E	72°13' N	250	(*19*)
28	RUS17	Russia	Yakutia	Indigirka River	148°00' E	69°00' N	150	unpublished
29	ALT01	Russia	Altai	Mongun	90°18' E	50°18' N	2300	(*22*)
30	ALT02	Russia	Altai	Mongun	90°18' E	50°18' N	2300	(*22*)
31	MON09	Mongolia	Archangai	Khorgo	99°52' E	48°10' N	2064	(*23*)
32	MON03	Mongolia	Övörkhangai	Uurgat	101°46' E	46°40' N	2100	(24)
33	MON05	Mongolia	Övörkhangai	Uurgat	101°46' E	46°40' N	2100	(24)
34	PAK04	Pakistan	Satpara Valley	Site2	75°30' E	35°10' N	3700	(*25*)
35	CHI01	China	Qinghai	Delingha	97°40' E	37°27' N	4019	(*26*)
36	CHI01	China	Qinghai	Delingha	97°40' E	37°27' N	4019	(*26*)
37	TIB01	China	Qamdo	QS9	97°08' E	31°07' N	4000	(*27*)
38	JAP01	Japan	Kagoshima	Yakushima Island	130°30' E	30°20' N	885	(*28*)
39	TAS01	Australia	West Coast	SRT	145°14' E	41°45' S	150	unpublished
40	DAR01	N. Zealand	Northland	Dargaville	173°48' E	35°55' S	40	(*29*)
41	DAR02	N. Zealand	Northland	Dargaville	173°48' E	35°55' S	40	(*29*)
42	DAR06	N. Zealand	Northland	Dargaville	173°48' E	35°55' S	40	(*29*)
43	DAR07	N. Zealand	Northland	Dargaville	173°48' E	35°55' S	40	(*29*)
44	NEW01	N. Zealand	Southland	Weka Farm	171°38’ E	42°30’ S	110	unpublished
FL	ICE01	Iceland	Katla	Drumbabót	19°45' W	63°41' N	50	(*21 main text*)
FL	Chang	N. Korea	Changbaishan	Xiaoshahe	127°58' E	42°02' N	2100	(*22 main text*)

Supplementary Table 2. Tree-ring network. Continuation of Table S1 (No. = 1–44), with AR zone referring to the atmospheric radiocarbon zones (*17*) (Supplementary Fig. S1), sample size providing the number of individual trees (t) and samples (s) covering the intervals 770–780 or 990–1000 CE, and the climate signal denoting the predominant growth controlling meteorological parameter (temperature, mixed or hydroclimate).

No	Sample material	Event	AR zone	Genus	Specific epithet	Sample size	Climate signal	Data contributor
1	woodblock	774	NHZ0	*Tsuga*	*mertensiana*	13t	temp	G. Wiles
2	woodblock	774	NHZ1	*Tsuga*	*mertensiana*	12t	temp	G. Wiles
3	woodblock	774	NHZ1	*Picea*	*mariana*	3t	temp	D. Arseneault
4	woodblock	774	NHZ1	*Pinus*	*ponderosa*	7t/12s	mixed	J. H. Speer
5	^{14}C values	774	NHZ2	*Pinus*	*longaeva*	25t/35s	mixed	A. J. T. Jull
6	woodblock	993	NHZ2	*Pseudotsuga*	*menziesii*	10t/21s	hydro	H. Grissino-Mayer
7	woodblock	774	NHZ2	*Pseudotsuga*	*menziesii*	10t/21s	hydro	H. Grissino-Mayer
8	split wood	774	SHZ1-2	*Austrocedrus*	*chilensis*	16s	hydro	D. A. Christi
9	split wood	774	SHZ1-2	*Fitzroya*	*cupressoides*	46s	temp	A. Lara
10	split wood	993	SHZ1-2	*Fitzroya*	*cupressoides*	56s	temp	A. Lara
11	split cellulose	993	NHZ0	*Pinus*	*sylvestris*	3t	temp	N. Loader
12	woodblock	993	NHZ0	*Pinus*	*sylvestris*	60-70t	temp	H. Grudd
13	split cellulose	774	NHZ0	*Pinus*	*sylvestris*	3t	temp	N. Loader
14	split cellulose	993	NHZ0	*Pinus*	*sylvestris*	3t	temp	N. Loader
15	woodblock	774	NHZ0	*Picea*	*abies*	10t	temp	H. W. Linderholm
16	woodblock	993	NHZ0	*Picea*	*abies*	10t	temp	H. W. Linderholm
17	woodblock	774	NHZ1	*Quercus*	*spp*		hydro	L. Wacker
18	woodblock	774	NHZ1	*Abies*	*alba*	75t	hydro	F. Herzig
19	woodblock	774	NHZ1	*Pinus*	*cembra*	26s	temp	K. Nicolussi
20	woodblock	774	NHZ1	*Pinus*	*cembra*	26s	temp	K. Nicolussi
21	woodblock	774	NHZ1	*Larix*	*decidua*		temp	L. Wacker
22	woodblock	774	NHZ1	*Picea*	*abies*		temp	M. Bernabei
23	woodblock	774	NHZ1	*Pinus*	*heldreichii*	2t	mixed	A. Seim
24	woodblock	774	NHZ1	*Pinus*	*heldreichii*	9t	mixed	P. J. Krusic
25	woodblock	774	NHZ0	*Larix*	*sibirica*		temp	R. Hantemirov
26	^{14}C values	774	NHZ0	*Larix*	*sibirica*	20t	temp	A. J. T. Jull
27	woodblock	774	NHZ0	*Larix*	*gmelinii*	10–15t	temp	A. Kirdyanov
28	woodblock	774	NHZ0	*Larix*	*cajanderi*	24t	temp	O. (S.) Churakova
29	woodblock	774	NHZ1	*Larix*	*sibirica*	26t	temp	V. Myglan
30	woodblock	993	NHZ1	*Larix*	*sibirica*	26t	temp	V. Myglan
31	woodblock	774	NHZ1	*Pinus*	*sibirica*		hydro	N. Pederson
32	woodblock	774	NHZ1	*Larix*	*sibirica*	39s	hydro	A. Hessl
33	woodblock	993	NHZ1	*Larix*	*sibirica*	39s	hydro	A. Hessl
34	woodblock	774	NHZ2	*Juniperus*	*excelsa*	8t	mixed	J. Esper
35	woodblock	774	NHZ2	*Juniperus*	*przewalskii*	198t	mixed	B. Yang
36	woodblock	993	NHZ2	*Juniperus*	*przewalskii*	198t	mixed	B. Yang
37	woodblock	774	NHZ2	*Juniperus*	*tibetica*		temp	A. Bräuning
38	woodblock	774	NHZ2	*Cryptomeria*	*japonica*	12t	mixed	F. Miyake
39	woodblock	774	SHZ1-2	*Lagarostrobos*	*franklinii*		unknown	E. Cook
40	woodblock	774	SHZ1-2	*Agathis*	*australis*	11t	mixed	G. Boswijk
41	woodblock	993	SHZ1-2	*Agathis*	*australis*	11t	mixed	G. Boswijk
42	woodblock	774	SHZ1-2	*Agathis*	*australis*	11t	mixed	G. Boswijk
43	woodblock	774	SHZ1-2	*Agathis*	*australis*	11t	mixed	G. Boswijk
44	woodblock	774	SHZ1-2	*Manoao*	*colensoi*		mixed	J. Palmer
FL	woodblock	774	NHZ0	*Betula*	*pubescens*		temp	Ó. Eggertsson
FL	woodblock	774	NHZ1	*Larix*	*spp*		temp	C. Oppenheimer

Supplementary Table 3. Cellulose extraction. Workflow of the different processing steps that were necessary for ^{14}C measurements at the Laboratory of Ion Beam Physics at ETH Zurich, Switzerland (Methods).

Workflow	1	2	3	4	5	6
**Process**	Splitting annual tree rings	Mercerization	Acidifying	Basifying	Acidifying and bleaching to cellulose	Freeze-drying
**Time (h)**		12.0	1.0	1.5	2.5	overnight
**Reagent**		4% NaOH	4% HCl	4% NaOH	4% HCl + 5% $NaClO_4$	

Supplementary Table 4. Model evidence. ^{14}C increase ($\Delta\Delta^{14}C$) and the modelled additional ^{14}C production (best fit) are given in the first two columns for both event. The increase from the pure measurement is given in the third column. The last two columns show the absolute and relative mean ^{14}C concentration ($\Delta^{14}C$) for each 11-year block (* average (771,772,773) / average (775,776,777); ** average (770:780); *** compared to NH mean). No data were available (na).

774

	Modelled $\Delta\Delta^{14}C$ (‰)			Modelled production atom (10^{25})			Increase* $\Delta\Delta^{14}C$ (‰)			11–year average $\Delta^{14}C$**(‰)			Offset $\Delta\Delta^{14}C$***(‰)		
NH mean	15.58	±	0.54	103.4	+<br>–	4.3<br>3.1	15.9	±	0.7	–10.47	±	0.36	na		na
NH0	15.2	±	1.08	100.9	+<br>–	9.2<br>7.4	16.8	±	0.5	–8.87	±	0.55	1.6	±	0.66
NH1	16.34	±	0.71	108.4	+<br>–	6.8<br>4.9	14.4	±	0.6	–10.67	±	0.38	–0.2	±	0.52
NH2	14.06	±	0.87	93.3	+<br>–	8<br>5.5	14.6	±	0.6	–11.69	±	0.51	–1.22	±	0.63
SH	14.01	±	0.75	88.3	+<br>–	5.5<br>6.1	14.6	±	0.6	–14.51	±	0.22	–4.04	±	0.42

993

	Modelled $\Delta\Delta^{14}C$ (‰)			Modelled production atom (10^{25})			Increase* $\Delta\Delta^{14}C$ (‰)			11–year average $\Delta^{14}C$**			Offset $\Delta\Delta^{14}C$***		
NH	8.51	±	0.99	55.5	+<br>–	7.24<br>6.03	9	±	0.6	–14.02	±	0.53	na		na
SH	8.52	±	1.51	53	+<br>–	10.84<br>8.43	8.8	±	1	–17.49	0	0.55	–3.48	±	0.76

Supplementary Table 5. Ice core data. Quasi-independent ^{10}Be evidence of the two radiometric time markers in the 770s and 990s from five high-resolution ice core measurements in Greenland and Antarctica[23,24] (Fig. 1). The accumulation rate (m water equivalent year^{-1}) of the individual records ranges from 0.027–0.210 (DF01 to NEEM). Please further note that the DF01 time period is based on $^{10}Be/^{14}C$ matching (*).

**Ice Core**	**Location**	**Depth Interval (m)**	**Time Period**	**Citation**
NEEM(2011-S1)	77.45N/51.06W	275.62-284.35	760-802 CE	(*24*)
NEEM(2011-S1)	77.45N/51.06W	275.62-284.35	989-1001 CE	(*24*)
TUNU2013	78.04N/33.88W	156.48-157.41	770-777 CE	(*24*)
NGRIP	75.12N/42.32W	244.75-249.70	773-800 CE	(*24*)
WDC	79.42S/120.09W	295.00-305.00	770-809 CE	(*24*)
DF01	77.37S/39.70E	57.53-58.60	763-794 CE*	(*23*)

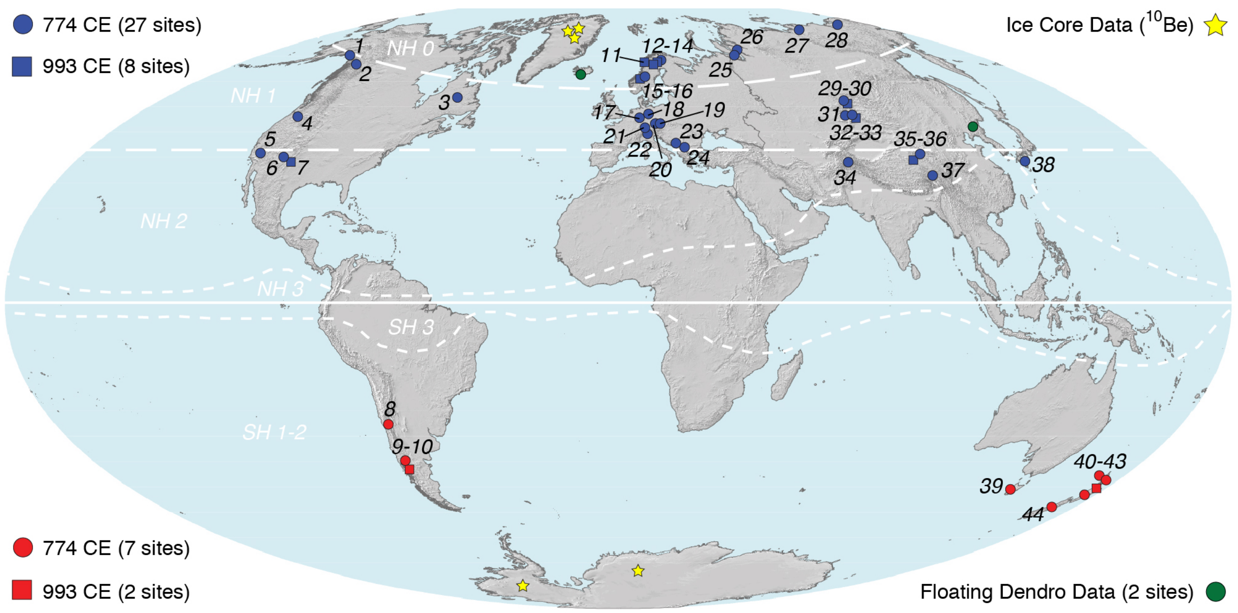

Supplementary Fig. 1. COSMIC network. Distribution of 44 tree-ring records from which cellulose was extracted for annual ^{14}C measurements during the intervals 770–780 and 990–1000 CE (circles and rectangles). Independent ^{14}C evidence from two floating tree-ring chronologies[21,22] (green), and five (quasi) annually resolved ice-core ^{10}Be records[23,24] (yellow). White dashed lines refer to atmospheric radiocarbon zones[17]. All data are chronologically ordered from no. 1–44 (Supplementary Tables 1-2). The map reflects knowledge from the authors and was created via software ArcGIS 10.1 SP1 for Desktop by Esri.

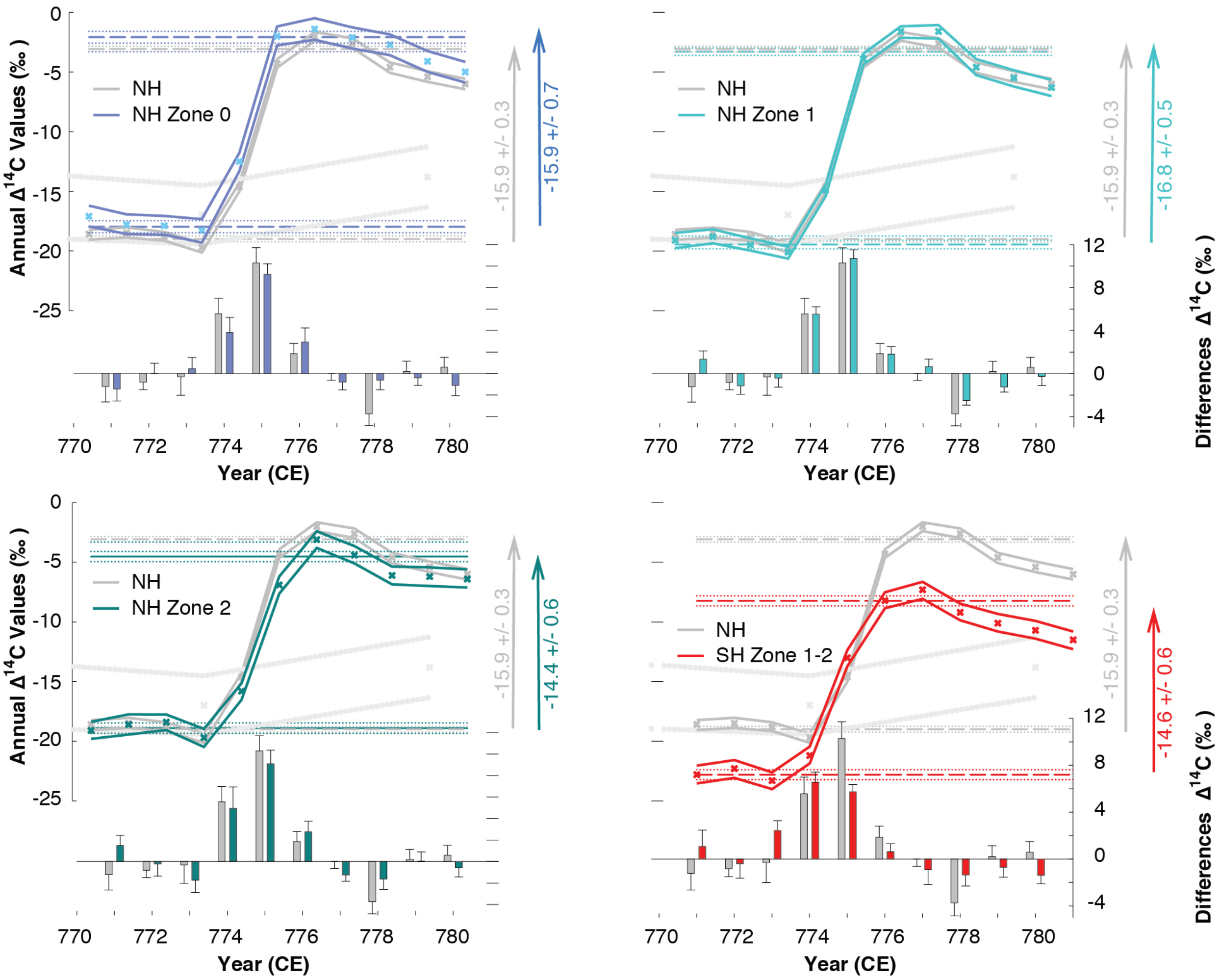

Supplementary Fig. 2. Radiocarbon zones. Annual ^{14}C content of the individual tree rings formed between 770 and 780 CE and separated into the meridional atmospheric ^{14}C zones[17].

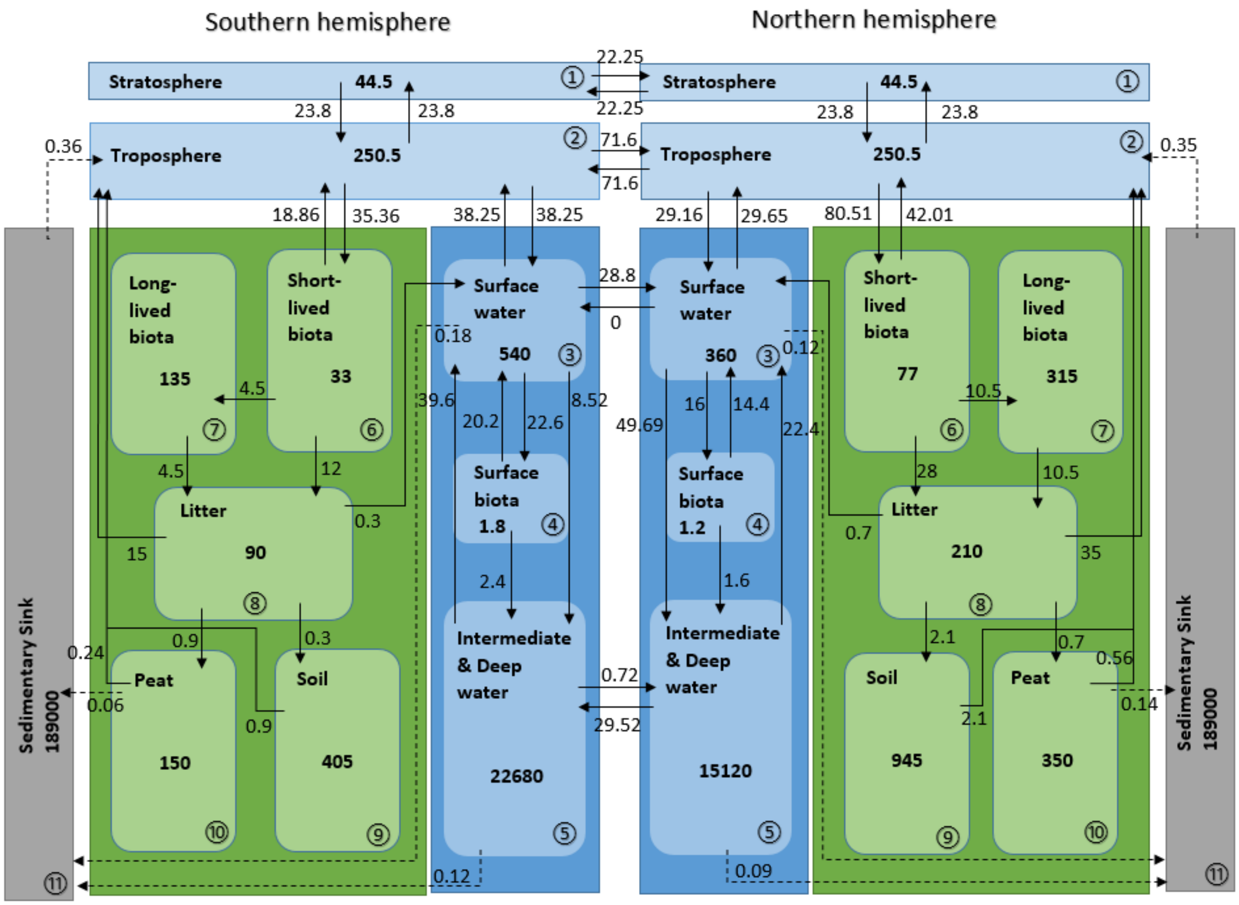

Supplementary Fig. 3. Global carbon box model. Diagram of Earth's pre-industrial carbon-cycle modified from[12]. Shown are major reservoirs (circled no. 1–11), together with their masses (bold values), as well as annual fluxes between the reservoirs. Inventory units are reported in 10^{12} kg, carbon fluxes are given in 10^{12}kg.yr^{-1}.

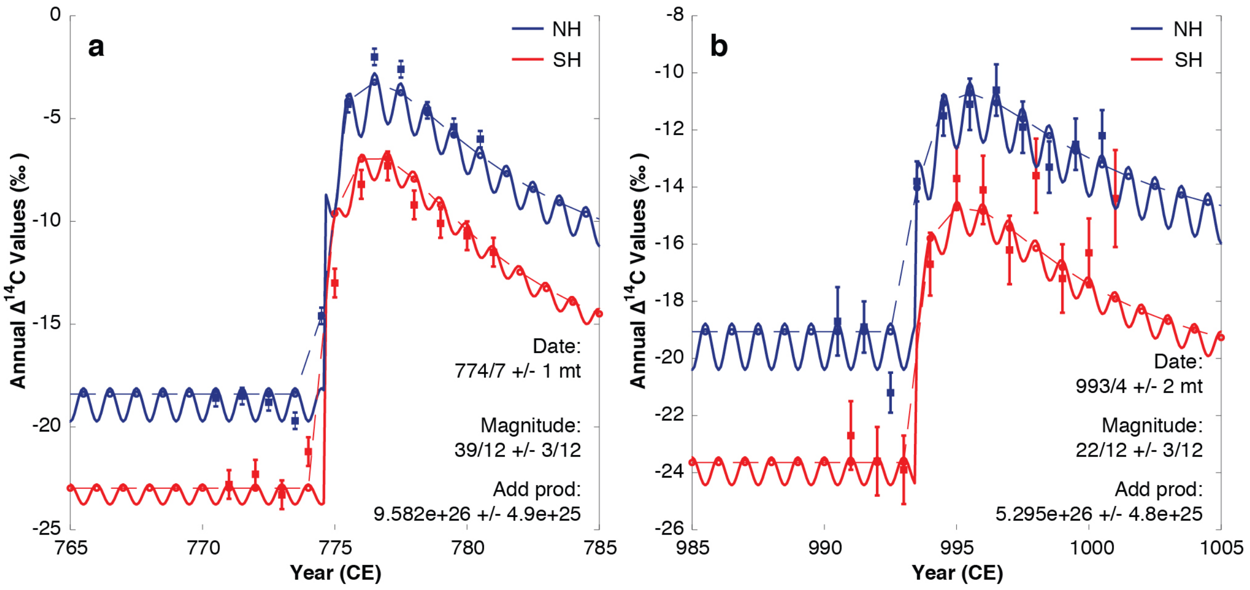

Supplementary Fig. 4. Model evidence. Solid squares give the measured mean radiocarbon concentrations for the NH (blue) and the SH (red), when simulating both, the events in 774 and 993 CE (**a, b**). The solid line shows the modelled atmospheric radiocarbon concentration while the open circles are the modelled radiocarbon concentrations expected from the seasonal uptake of atmospheric CO_2 by trees fitted to the measurements of the SH and the NH.

Supplementary References

1. Wiles, G.C. et al. Surface air temperature variability reconstructed with tree rings for the Gulf of Alaska over the past 1200 years. *Holocene* **24**, 198–208 (2014).
2. Gennaretti, F., Arseneault, D., Nicault, A., Perreault, L. & Bégin, Y. Volcano-induced regime shifts in millennial tree-ring chronologies from northeastern North America. *Proc. Natl. Acad. Sci. U.S.A.* **111**, 10077–10082 (2014).
3. Clark, P.W., Speer, J.H. & Winship, L.J. Identifying and separating Pandora Moth outbreaks and climate from a 1500-year Ponderosa Pine chronology from Central Oregon. *Tree-Ring Res.* **73**, 113–125 (2017).
4. Salzer, M.W., Hughes, M.K., Bunn, A.G. & Kipfmueller, K.F. Recent unprecedented tree-ring growth in bristlecone pine at the highest elevations and possible causes. *Proc. Natl. Acad. Sci. U.S.A.* **106**, 20348–20353 (2009).

5. Grissino-Mayer, H.D. *Tree-ring reconstructions of climate and fire history at El Malpais National Monument, New Mexico*, University of Arizona, PhD dissertation (1995).

6. LeQuesne, C., Acuña, C., Boninsegna, J.A., Rivera, A. & Barichivich, J. Long-term glacier variations in the Central Andes of Argentina and Chile, inferred from historical records and tree-ring reconstructed precipitation. *Palaeogeogr. Palaeoclimatol. Palaeoecol.* **281**, 334–344 (2009).

7. Lara, A. & Villalba, R. A 3620-year temperature record from *Fitzroya cupressoides* tree rings in southern South America. *Science* **260**, 1104–1106 (1993).

8. Young, G. et al. Changes in atmospheric circulation and the Arctic Oscillation preserved within a millennial length reconstruction of summer cloud cover from northern Fennoscandia. *Clim. Dyn.* **39**, 495–507 (2012).

9. Loader, N.J., Young, G.H.F., Grudd, H. & McCarroll, D. Stable carbon isotopes from Torneträsk, northern Sweden provide a millennial length reconstruction of summer sunshine and its relationship to Arctic. *Quat. Sci. Rev.* **62**, 97–113 (2013).

10. Grudd, H. et al. A 7400-year tree-ring chronology in northern Swedish Lapland: natural climatic variability expressed on annual to millennial timescales. *Holocene* **12**, 657–665 (2002).

11. Gunnarson, B.E. Temporal distribution pattern of subfossil pines in central Sweden: perspective on Holocene humidity fluctuations. *Holocene* **18**, 569–577 (2008).

12. Büntgen, U., Esper, J., Frank, D.C., Nicolussi, K. & Schmidhalter, M. A 1052-year tree-ring proxy for Alpine summer temperatures. *Clim. Dyn.* **25**, 141–153 (2005).

13. Nicolussi, K. et al. A 9111-year-long conifer tree-ring chronology for the European Alps: a base for environmental and climatic investigations. *Holocene* **19**, 909–920 (2009).

14. Seim, A. et al. Climate sensitivity of a millennium-long pine chronology from Albania. *Clim. Res.* **51**, 217–228 (2012).

15. Klippel, L. et al. High-elevation inter-site differences in Mount Smolikas tree-ring width data. *Dendrochronologia* **44**, 164–173 (2017).

16. Hantemirov, R.M. & Shiyatov, S.G. A continuous multi-millennial ring-width chronology in Yamal, northwestern Siberia. *Holocene* **12**, 717–726 (2002).

17. Briffa, K.R. et al. Reassessing the evidence for tree-growth and inferred temperature change during the Common Era in Yamalia, northwest Siberia. *Quat. Sci. Rev.* **72**, 83–107 (2013).

18. Briffa, K.R. et al. Trends in recent temperature and radial tree growth spanning 2000 years across northwest Eurasia. *Phil. Trans. R. Soc. B.* **363**, 2271–2284 (2008).

19. Naurzbaev, M.M., Vaganov, E.A., Sidorova, O.V. & Schweingruber, F.H. Summer temperatures in eastern Taimyr inferred from a 2427-year late-Holocene tree-ring chronology and earlier floating series. *Holocene* **12**, 727–736 (2002).

20. Hughes, M.K., Vaganov, E.A., Shiyatov, S., Touchan, R. & Funkhouser, G. Twentieth-century summer warmth in northern Yakutia in a 600-year context. *Holocene* **9**, 629–634 (1999).

21. Sidorova, O.V. & Naurzbaev, M. Response of Larix cajanderi to climatic changes at the upper timberline and flood-plane terrace from Indigirka River valley. *Russian Forest Management* **2**, 73–75 (2002).

22. Myglan, V, Oidupaa, O.C., Kirdyanov, A. & Vaganov, E.A. 1929-year tree-ring chronology for the Altai–Sayan region (Western Tuva). *Archaeol. Ethnol. Anthropol. Eurasia* **36**, 25–31 (2008).

23. Pederson, N., Hessl, A.E., Baatarbileg, N., Anchukaitis, K.J. & Di Cosmo, N. Pluvials, droughts, the Mongol Empire, and modern Mongolia. *Proc. Natl. Acad. Sci. U.S.A.* **111**, 4375–4379 (2014).

24. Hessl, A.E. et al. Past and future drought in Mongolia. *Sci. Adv.* **4**, e1701832 (2018).

25. Esper, J., Frank, D.C., Wilson, R.J., Büntgen, U. & Treydte, K. Uniform growth trends among central Asian low-and high-elevation juniper tree sites. *Trees* **21**, 141–150 (2007).

26. Yang, B. et al. A 3,500-year tree-ring record of annual precipitation on the northeastern Tibetan Plateau. *Proc. Natl. Acad. Sci. U.S.A.* **111**, 2903–2908 (2014).

27. Bräuning, A. Climate history of the Tibetan Plateau during the last 1000 years derived from a network of Juniper chronologies. *Dendrochronologia* **19**, 127–137 (2001).

28. Kitagawa, H. & Matsumoto, E. Climatic implications of $\delta^{13}C$ variations in a Japanese cedar (*Cryptomeria japonica*) during the last two millennia. *Geophys. Res. Lett.* **22**, 2155–2158 (1995).

29. Boswijk, G. et al. The late Holocene kauri chronology: assessing the potential of a 4500-year record for palaeoclimate reconstruction. *Quat. Sci. Rev*. **90**, 128–142 (2014).
